# The Role of Norepinephrine and Its α-Adrenergic Receptors in the Pathophysiology and Treatment of Major Depressive Disorder and Schizophrenia: A Systematic Review

**DOI:** 10.3389/fpsyt.2017.00042

**Published:** 2017-03-17

**Authors:** Vladimir Maletic, Anna Eramo, Keva Gwin, Steve J. Offord, Ruth A. Duffy

**Affiliations:** ^1^Department of Neuropsychiatry and Behavioral Science, University of South Carolina, Columbia, SC, USA; ^2^Medical Affairs – Psychiatry, Lundbeck LLC, Deerfield, IL, USA; ^3^Medical Affairs, Otsuka Pharmaceutical Development and Commercialization, Inc., Princeton, NJ, USA

**Keywords:** norepinephrine, α-adrenergic receptors, major depressive disorder, schizophrenia, valence systems, pathophysiology, antidepressants, antipsychotics

## Abstract

Norepinephrine (NE) is recognized as having a key role in the pathophysiology of major depressive disorder (MDD) and schizophrenia, although its distinct actions *via* α-adrenergic receptors (α-ARs) are not well defined. We performed a systematic review examining the roles of NE and α-ARs in MDD and schizophrenia. PubMed and ProQuest database searches were performed to identify English language papers published between 2008 and 2015. In total, 2,427 publications (PubMed, *n* = 669; ProQuest, *n* = 1,758) were identified. Duplicates, articles deemed not relevant, case studies, reviews, meta-analyses, preclinical reports, or articles on non-target indications were excluded. To limit the review to the most recent data representative of the literature, the review further focused on publications from 2010 to 2015, which were screened independently by all authors. A total of 16 research reports were identified: six clinical trial reports, six genetic studies, two biomarker studies, and two receptor studies. Overall, the studies provided indirect evidence that α-AR activity may play an important role in aberrant regulation of cognition, arousal, and valence systems associated with MDD and schizophrenia. Characterization of the NE pathway in patients may provide clinicians with information for more personalized therapy of these heterogeneous diseases. Current clinical studies do not provide direct evidence to support the role of NE α-ARs in the pathophysiology of MDD and schizophrenia and in the treatment response of patients with these diseases, in particular with relation to specific valence systems. Clinical studies that attempt to define associations between specific receptor binding profiles of psychotropics and particular clinical outcomes are needed.

## Introduction

Major depressive disorder (MDD) and schizophrenia affect 151.2 and 26.3 million people worldwide, respectively, and are associated with high rates of morbidity and mortality (1). These disorders exert a large mental health burden and socioeconomic cost across the world, accounting for 24.5 and 5.3%, respectively, of disability-adjusted life-years attributed to all mental, neurological, and substance use disorders (2). The catecholamine neurotransmitter, norepinephrine (NE), has been speculated as having a role in depressive disorders since the 1950s (3), and schizophrenia since the 1970s (4), although a full understanding of the actions of NE in the pathophysiology of these diseases remains unclear.

**Figure 1:**
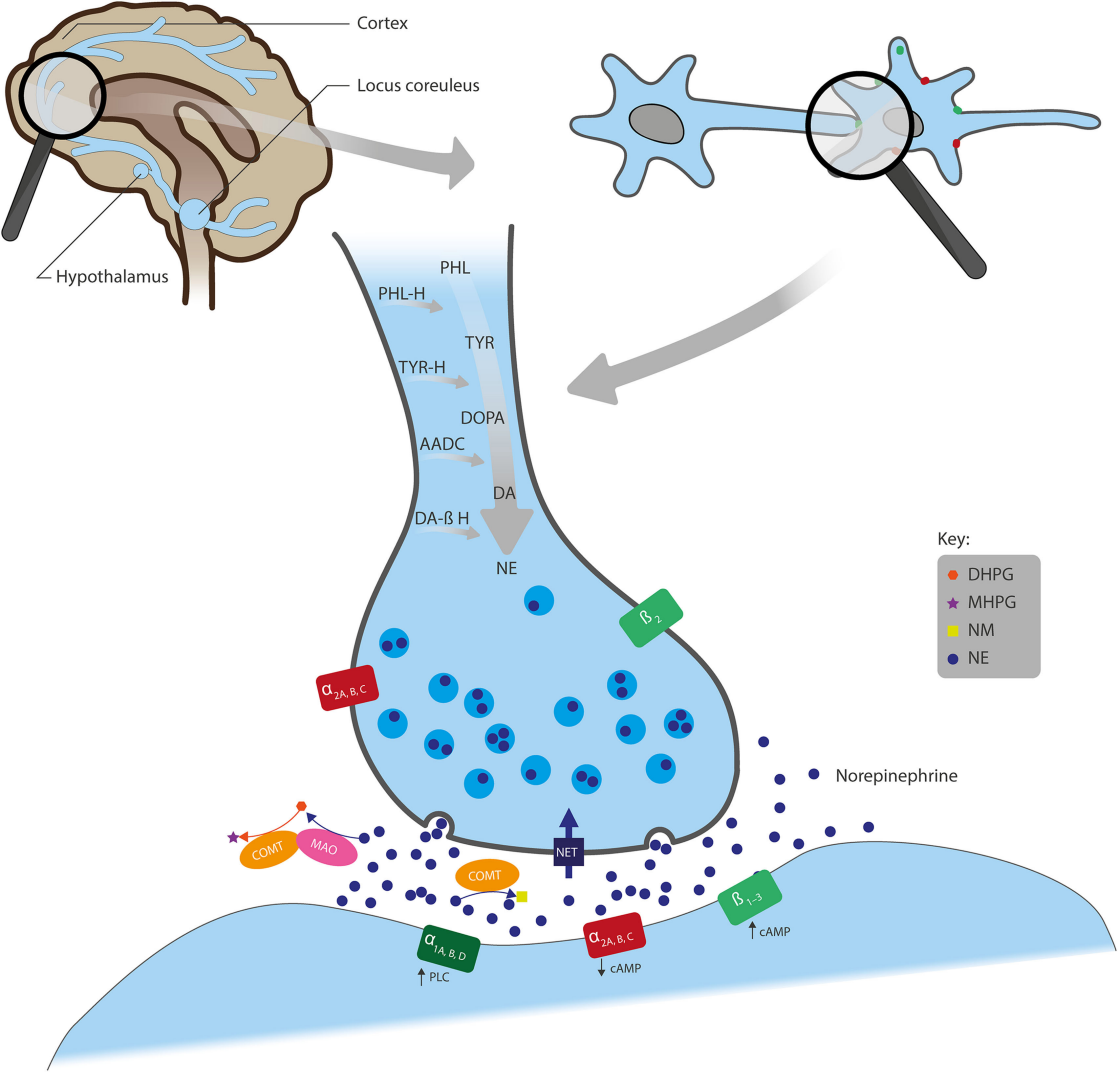
**Overview of the actions of NE in neural synapses**. Noradrenergic neurons originate from the locus coeruleus and project to regions of the forebrain, including the cortex and hypothalamus. NE is synthesized from the amino acids TYR and PHL and converted first to DOPA, DA, and further to NE by the enzymes TYR-H, AADC, and DA β-H, respectively, after which NE is stored in presynaptic vesicles. Following its release into the synaptic cleft, NE exerts its effects through binding to the adrenergic receptors (ARs): α_1A_, α_1B_, and α_1D_; α_2A_, α_2B_, and α_2C_; or β_1_, β_2_, and β_3_. α_1_- and β-ARs have a stimulatory effect on cell signaling, whereas α_2_-ARs inhibit signaling. Also, while ARs are mainly located post-synaptically, α_2_- and β_2_-AR subtypes can also be localized pre-synaptically. NE is removed from the synaptic cleft by either reuptake *via* NET (expressed on the presynaptic terminals of NE neurons and glial cells), inactivation through the catabolic enzyme COMT to NM, or metabolism by MAO into several transitional metabolites, including its principal brain metabolite, MHPG. AADC, l-aromatic amino acid decarboxylase; cAMP, cyclic adenosine monophosphate; COMT, catechol O-methyltransferase; DA, dopamine; DA β-H, dopamine β-hydroxylase; DHPG, dihydroxyphenylglycol; DOPA, 3,4-dihydroxyphenylalanine; MAO, monoamine oxidase; MHPG, 3-methoxy-4-hydroxyphenylglycol; NE, norepinephrine; NET, NE transporter; NM, normetanephrine; PHL, phenylalanine; PHL-H, phenylalanine hydroxylase; PLC, phospholipase C; TYR, tyrosine; TYR-H, tyrosine hydroxylase.

An overview of the synthesis, actions, and metabolism of NE is shown in Figure 1. NE exerts its effects through binding to G-protein coupled α- and β-adrenergic receptors (ARs). α-ARs are further divided into α_1_ and α_2_, and each of these has three subtypes: α_1A_, α_1B_, and α_1D_; α_2A_, α_2B_, and α_2C_. β-ARs include β_1_, β_2_, and β_3_ subtypes (5). Generally, α_1_- and β-ARs have a stimulatory effect on cell signaling, as they have been shown to increase intracellular phospholipase C or cyclic adenosine monophosphate (cAMP), respectively, whereas α_2_-ARs suppress intracellular cAMP and generally have an inhibitory influence on signaling (Figure 1) (6). NE has the highest affinity for α_2_-ARs (5) so low-level NE release may inhibit neuronal activity, whereas increased neural transmission arising from NE binding to stimulatory α_1_- and β-ARs only occurs at higher NE concentrations (7).

Noradrenergic receptors are found on nerve fibers that originate from the locus coeruleus (LC) and project to many parts of the forebrain, including the cortex, cerebellum, amygdala, hippocampus, basal ganglia, thalamus, and hypothalamus (Figure 1) (8). Noradrenergic heteroreceptors are also located on glutamate, gamma-aminobutyric acid (GABA), dopamine (DA), serotonin (5-HT), histamine, and orexin neurons, as well as in glial and immune cells. Therefore, in addition to being autoregulated by presynaptic α_2A_-ARs, α_2C_-ARs (5), and β_2_-ARs (9), NE signaling is also regulated by other neurotransmitters, such as inhibitory GABA and excitatory glutamate (3, 10). Taken together, this suggests that NE receptors within these pathways play a role in a broad range of brain functions, such as arousal, stress response, memory consolidation, immune response, endocrine function, sleep/wakefulness, and pain-threshold regulation (11).

The Research Domain Criteria (RDoC) matrix was created to help identify brain mechanisms that explain the pathology of psychiatric disorders, improve accuracy of diagnosis, and predict responses to treatment (12). The RDoC matrix classifies symptoms into negative valence systems (including fear and anxiety), positive valence systems (including motivation and reward-seeking behavior), cognitive systems (including attention, perception, declarative, and working memory), social processing systems (including affiliation and attachment), and arousal/regulatory systems (including circadian rhythms and sleep) (13). By including genetic and other factors that influence neurotransmission, the RDoC matrix provides a more comprehensive model of psychiatric diseases, including MDD and schizophrenia.

Major depressive disorder is mostly characterized by a depressed mood, fatigue, a diminished ability to think or concentrate, and disruptions to sleep/wakefulness, circadian rhythms, and immune responses (14). Evidence has shown that these symptoms can be influenced by NE activity in the LC *via* α-AR modulation. Two of the first antidepressant treatments (ADTs) were iproniazid, which inhibits monoamine oxidase (MAO), and imipramine, which blocks reuptake of serotonin and NE, leading to increased concentrations of these neurotransmitters (15). NE activity in the LC has been shown to be altered in patients with MDD compared with controls: histopathology studies have suggested patients with MDD have increased levels of tyrosine hydroxylase and a reduced density of NE transporter (NET) in the LC (16), the latter confirmed by radioligand binding studies (17). Concentrations of 3-methoxy-4-hydroxyphenylglycol (MHPG), the major NE metabolite, in the cerebrospinal fluid (CSF) have been shown to positively correlate with lifetime mood burden, a composite measure reflecting the number, duration, and intensity of depressive episodes (18). Moreover, salivary MHPG levels in men were recently shown to correlate with depressive symptom scores (19).

Desensitized α_1_-ARs in the brains of depressed patients have previously been identified (20). Conversely, studies have shown that both the affinity and density of inhibitory α_2_-ARs are increased in the LC and prefrontal cortex of patients with MDD compared with controls (21, 22), which may reflect a compensatory response related to high NE levels. However, blocking α_2_-ARs using yohimbine has been shown to improve memory consolidation in patients with MDD, suggesting that increased α_2_-AR density may also have detrimental effects in these patients (23). MDD could, therefore, be conceptualized as a highly phenotypically and biologically heterogeneous condition, whereby depressed patients may experience both under- and over-arousal that may vary regionally (24).

Like MDD, schizophrenia is a heterogeneous disease where symptomatology includes positive (e.g., paranoid delusions, auditory hallucinations, incoherent thinking), negative (e.g., affective blunting, inactivity, impoverished speech), affective, and cognitive symptoms that can vary independently in each patient (4). In general, positive symptoms are aggravated by selective, indirect NE agonists such as yohimbine, and ameliorated by functional NE antagonists such as clonidine and oxypertine (4). Furthermore, adjunctive ADTs that alter NE activity (e.g., duloxetine) have been shown to relieve negative symptoms (25), suggesting NE may have a role in the pathophysiology of schizophrenia. However, the heterogeneity of the disease is reflected in postmortem studies, which have shown that brain concentrations of NE in patients with schizophrenia vary across populations (4).

An increased understanding of the involvement of α-ARs in the pathophysiology of MDD and schizophrenia is reflected in the pharmacology of recently approved ADTs and antipsychotics (APs). Many treatments for MDD act on overall NE levels (including uptake and MAO inhibitors) or on α-ARs (15). More recent therapies include selective NE reuptake inhibitors, such as reboxetine (26); NE and DA uptake inhibitors, such as bupropion (27); mixed serotonergic and noradrenergic reuptake inhibitors, such as venlafaxine, duloxetine, milnacipran, levomilnacipran, and desvenlafaxine (15); the selective serotonin reuptake inhibitor (SSRI) and 5-HT_1A_ partial agonist vilazodone (28); multimodal agents, such as vortioxetine, a SSRI, 5-HT_1A_ agonist, 5-HT_1B_ partial agonist, and 5-HT_1D_, 5-HT_3_, and 5-HT_7_ antagonist (29); and compounds that block α_2_-ARs and 5-HT_2_ receptors and increase activity at 5-HT_1_ receptors, such as mirtazapine (30). Many of these agents either directly or indirectly modulate NE.

Although most current APs are DA D_2_ receptor antagonists or partial agonists/antagonists, several second generation APs also act on other neurotransmitter receptors, including α-ARs (31). In schizophrenia, it is generally thought that blocking α_1_-ARs suppresses positive symptoms, while blocking α_2_-ARs relieves negative and cognitive symptoms (31). Indeed, clozapine, a prototypical second generation AP whose mechanism of action is not fully understood and has not yet been replicated by any other agent, is known to be an antagonist of α_1_-ARs with higher affinity for these receptors than for D_2_ receptors, as well as acting as an antagonist of α_2_-ARs (32). Administration of clozapine in rats increases α_1_-AR density in the cortex and thalamus (33). While clozapine results in superior efficacy in treatment-resistant schizophrenia and reduced extrapyramidal symptoms compared with standard APs and other second generation APs (34), its ability to induce potentially fatal agranulocytosis restricts its use clinically (32). Other APs such as risperidone, olanzapine, quetiapine, aripiprazole, asenapine, lurasidone, and cariprazine were subsequently developed in an attempt to improve efficacy and side effect profiles (32). Indeed, risperidone is a potent α-AR antagonist (35). Moreover, the second generation AP brexpiprazole has a distinct pharmacologic profile with purported serotonin-DA modulating activity and α-AR antagonist activity (36). In phase II and III studies, brexpiprazole was efficacious in the treatment of patients with schizophrenia (37), and when administered adjunctively in patients with MDD and an inadequate response to ADTs (38). Therefore, it is of considerable interest to understand the role that NE antagonism may play in ameliorating the symptoms of MDD and schizophrenia.

This systematic review will focus on the role of α-ARs in the pathophysiology of MDD and schizophrenia, as well as in the mechanism of action of compounds used in clinical studies of these diseases. In particular, this review aims to assess how the action of these compounds at α-ARs may be related to the RDoC negative and positive valence systems, arousal, and cognition. The review will include only those compounds exerting their action on α-ARs receptors, and not β-ARs or NET, as the hope is to elucidate the role of α-ARs in the perceived efficacy of these compounds. Moreover, this review aims to establish whether the current clinical evidence is sufficient to link α-ARs to clinical treatments or outcomes of MDD and schizophrenia, with the understanding that the nature of these heterogeneous diseases may make it difficult to identify a single common thread that definitively establishes such a link. The review will also identify gaps in the literature that future investigations need to address.

## Materials and Methods

### Literature Searches

Two literature searches were performed on the role of NE and α-ARs in MDD and schizophrenia. A PubMed search was carried out on December 11, 2015 and a ProQuest search was carried out on December 18, 2015. Both searches were conducted using the following Medical Subject Heading (MeSH) term search string: “norepinephrine” OR “noradrenaline” OR “alpha adrenergic receptor(s)” OR “beta adrenergic receptor(s)” AND “schizophrenia” OR “major depressive disorder” AND “translational medicine” OR “immunity” OR “microglia” OR “astrocyte(s)” OR “inflammation” OR “gene(s)” OR “brain chemistry” OR “biomarker” OR “neuronal plasticity” OR “cognition” OR “arousal” OR “sleep” OR “psychopharmacology” OR “brain development.” The original searches were limited to the period January 2008–December 2015 and publications in the English language.

### Literature Review Results

**Figure 2:**
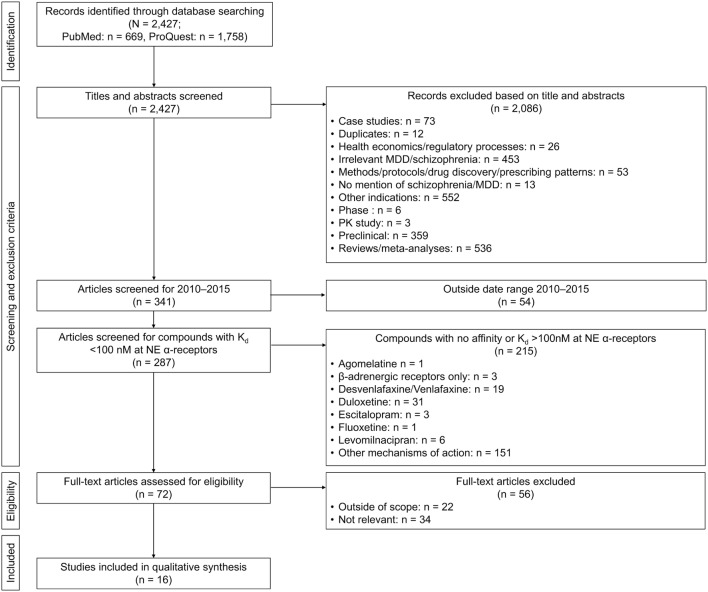
**Flow diagram of article selection**. MDD, major depressive disorder; NE, norepinephrine; PK, pharmacokinetics.

The searches yielded a total of 2,427 publications (PubMed, *n* = 669; ProQuest, *n* = 1,758). After exclusion of duplicates, articles deemed not relevant, case studies, reviews and meta-analyses, preclinical reports, or articles concerning other indications, abstracts, and full-length text were independently screened for eligibility by all the authors. To focus the review on the most recent information, it was decided that only articles published during the period January 2010–December 2015 would be included in full article screening. Psychotropics used in the treatment of MDD and schizophrenia do not generally exert a direct effect on β-ARs, with these receptors only influenced by a change in overall NE transmission. While some adverse events of psychotropics may be mediated by β-ARs, in order to focus the review on the role of NE related to the pathophysiology of psychiatric disorders, the review was limited to articles discussing the actions of NE at α-ARs only and on drugs with at least a moderate affinity for α-ARs (*K*_i_ < 100 nM). A total of 16 research reports were identified as meeting these inclusion criteria (Figure 2) including six clinical trial reports, six genetic studies, two biomarker studies, and two receptor studies (Table 1).

**Table 1:** **Summary of articles identified**.

Reference	Indication	Type of study
Chandley et al. (3)	MDD	Genetic
Houston et al. (45)		Genetic
Egami et al. (47)		Biomarker
Nyberg et al. (55)		PET study
Rivero et al. (48)		Receptor density
Kuffel et al. (40)		Clinical trial
Nagao et al. (41)		Clinical trial

Cheng et al. (49)	SCZ	Genetic
De Luca et al. (51)		Genetic
Liu et al. (50)		Genetic
Lochman et al. (53)		Genetic
Evers et al. (52)		Biomarker
Oranje and Glenthoj (39)		Clinical trial
Oranje and Glenthoj (42)		Clinical trial
Stenberg et al. (44)		Clinical trial
Terevnikov et al. (43)		Clinical trial

*MDD, major depressive disorder; SCZ, schizophrenia*.

## Results

Overall, we found that direct clinical evidence for the role of NE at α-ARs in the symptomatology of MDD and schizophrenia is limited. Six clinical trials were identified (three investigating clonidine and three investigating mirtazapine; one for each in MDD and two for each in schizophrenia), none of which were designed *a priori* to compare the effect of compounds with differing pharmacological profiles on outcomes of the study.

### Clinical Trials

#### Major Depressive Disorder

Clonidine, a specific α_2_-AR agonist, may suppress NE output, while simultaneously stimulating postsynaptic α_2_-ARs (39). In a study investigating the effects of clonidine in MDD patients and healthy controls, clonidine was shown to impair memory consolidation (word list learning) but did not affect working memory or memory retrieval (40). Conversely, mirtazapine, a noradrenergic and specific serotonergic ADT, selectively blocks α_2_-ARs and serotonin 5-HT_2_ and 5-HT_3_ receptors as well as histaminergic H_1_ receptors, and indirectly stimulates serotonin 5-HT_1A_ receptors (30). Mirtazapine was compared with the selective serotonin and norepinephrine reuptake inhibitor duloxetine and was shown to be superior in the reduction of Hamilton Rating Scale for Depression scores in patients with MDD (41). Therefore, caution may be needed when targeting α_2_-AR in order to balance effects on learning and memory consolidation, while effectively reducing depressive symptoms in patients with MDD.

#### Schizophrenia

Administration of clonidine to patients with schizophrenia increased pre-pulse inhibition of the startle reflex, a measure of sensorimotor gating known to be defective in schizophrenia, to levels similar to healthy controls, whereas inhibition of this reflex remained deficient in placebo-treated patients (39). Similarly, P50 suppression, a related measure of sensory gating, was reduced in patients with schizophrenia compared with healthy controls. However, clonidine increased P50 suppression in patients, thereby causing enhanced sensory gating and potentially improving cognitive abilities in these patients (42).

Patients treated with adjunctive mirtazapine showed an overall improvement in total Positive and Negative Syndrome Scale (PANSS) scores, predominantly driven by improvements in positive symptom scores (43), although this may have been due to its sedating properties. Further investigation into factors that may predict and mediate this response revealed that improvements were attributable to increased spatial visualization ability and motor skill as measured by the block design test. Furthermore, pathway analysis indicated that perceived perceptual/cognitive benefits, related to the improvement in attention and processing speed, may be attributable to mirtazapine’s antidepressant properties, rather than direct impact on cognition and negative symptoms of schizophrenia (44). One possible interpretation of these findings is that mirtazapine-associated modulation of α_1_-ARs and α_2_-ARs and consequent “tuning” of NE/5-HT signaling may have played a key role in its ameliorative effect on the positive symptoms of schizophrenia.

### Predicting Response to Treatment

Many investigations have been carried out to determine genetic or metabolic signatures associated with MDD and schizophrenia. As well as providing insights into the pathophysiology of these disorders, these reports may assist clinicians with predicting how patients will respond to therapy and providing more personalized care. We identified six reports investigating the genetic variants of noradrenergic receptors or proteins expressed within the noradrenergic synthetic pathway, and their relationship to either symptoms of MDD and schizophrenia or the side effects of treatment with agents that have activity at α-ARs. We also identified two studies investigating NE metabolites as biomarkers of MDD and schizophrenia, and two studies investigating the binding or distribution of NE-associated receptors or transporters.

#### Major Depressive Disorder

Locus coeruleus NE activity is non-specifically modulated by glutamatergic input, and so changes in glutamate levels could impact the effects of NE in this brain region. Analysis of genes related to glutamatergic function showed reduced expression in LC astrocytes of patients with MDD (3). Additionally, this study provides evidence of astrocyte dysfunction in the noradrenergic LC region in patients with MDD.

In another study, expression of *SLC6A2rs36024* [a single nucleotide polymorphism (SNP) of NET] was found to be associated with improvement of Montgomery–Åsberg Depression Rating Scale scores during olanzapine–fluoxetine treatment of patients (45). Olanzapine acts as an α_1_-AR antagonist with moderate affinity (46), which may in part explain its beneficial therapeutic response given dysfunctional reuptake by NET. Moreover, saliva levels of MHPG were shown to be higher in patients than controls, likely reflecting aberrant NE turnover and less efficient neurotransmission (47). These studies support targeting the reuptake of NE or antagonism of stimulatory α_1_-ARs for the treatment of patients with MDD.

Rivero et al. (2014) investigated the density of ARs in MDD (48). Using radioligand saturation binding experiments, α_2_- and β_1_-AR densities were found to be higher in the prefrontal cortex of ADT-free patients with MDD than controls, possibly as a compensation associated with altered NE turnover in MDD. In antidepressant-treated patients, α_2_-AR density remained increased over controls, whereas no difference was observed in β_1_-AR densities (48). These results suggest a resistance of α_2_-ARs to the downregulatory effect of ADTs, a potentially important consideration in the development of future ADTs. However, as the majority of brain tissue used in this study was collected from patients who committed suicide (48), these results also indicate that it is important to consider the role of α_2_-ARs in patients with MDD with respect to suicidality and treatment response.

#### Schizophrenia

Weight gain has been noted as a common side effect of second generation APs. We identified three genetic studies focusing on the association of genes encoding ARs and weight gain. Two reports provided evidence of a link between *ADRA1A* gene expression and increased weight gain, as well as body mass index during AP treatment (49, 50). Moreover, *ADRA1A* gene expression was positively associated with the cumulative number of metabolic syndrome components, with the presence of Arg347 allele of *ADRA1A* identified as a risk factor for metabolic abnormalities (49). Conversely, in nine genetic polymorphisms tested across genes coding for seven proteins related to noradrenergic receptors or NE synthesis or metabolism (*ADRA1A, ADRA2A, ADRA2C, ADRB3, DBH, MAOA*, and *COMT*), no association with AP-induced weight gain in patients with schizophrenia was identified (51).

Other identified articles provided evidence that genetic changes in NE metabolic markers, α-ARs, or other receptors that alter NE activity may influence relevant clinical characteristics of patients with schizophrenia. An investigation into metabolite biomarkers of schizophrenia revealed that levels of DA, homovanillic acid, NE, vanillylmandelic acid, and serotonin positively correlated with Full Scale Intelligence Quotient scores in patients with chromosome 22q11 deletion syndrome, which results in a higher risk of developing MDD, schizophrenia, and other psychiatric disorders (52). Another report discovered an interaction between an α_2A_-AR gene SNP and a specific methylenetetrahydrofolate reductase gene SNP, which encodes for an enzyme involved in DNA methylation, in patients with schizophrenia (53), suggesting a potential role for NE in the epigenetic control relevant to the pathogenesis of schizophrenia.

The overall pharmacological profile of a compound may play a role in its efficacy that may not initially be recognized. Like many APs, quetiapine binds to a broad range of receptors at varying affinities, including 5-HT_1A/2A/2C_, D_2_, histamine H_1_, and adrenergic α_1/2_ receptors (54). Quetiapine-extended release (XR) has been shown to also occupy NET in healthy participants, which may help contribute to its broad efficacy (55). Ziprasidone, another AP with affinity for NET and adrenergic α_1_-ARs, has also demonstrated efficacy as adjunctive treatment to SSRIs in treatment-resistant MDD (56) and anxious depression (57), although it is not approved for the treatment of MDD. Ziprasidone has also been effectively used as an adjunct in depressed patients who have had an inadequate response to escitalopram alone (58). However, it is unlikely that affinity for NET is necessary for the antidepressant effect of APs, as both olanzapine and clozapine have been shown to provide a greater relief of depressive symptoms in patients suffering from schizophrenia, compared to quetiapine (59).

## Discussion

Although it is known that NE and its α-ARs play key roles in the pathophysiology of MDD and schizophrenia, direct evidence from recent clinical reports is limited. This appears to be largely due to the trial designs used in most clinical studies. Indeed, there is a lack of direct comparisons of ADTs and APs with differing pharmacologic profiles that measure their impact on specific symptoms or disease outcomes. Overall, our review provided data illustrating that NE action at α-ARs can impact the RDoC negative valence systems and cognitive systems proposed to be involved in MDD and schizophrenia. We also identified several studies that suggested that not only can NE activity be used as a biomarker of MDD and schizophrenia, but that it may also be a useful tool for predicting patient response and adverse reactions related to pharmacological intervention.

### NE Effects on Negative Valence Systems

The RDoC negative valence system domain includes fear and anxiety (13), and can, along with disturbances in cognitive domain, be used to categorize some of the positive symptoms of schizophrenia, such as hallucinations (60). A biomarker study showed that elevated NE may be associated with cognitive disorganization, poor impulse control, suspiciousness, hostility, and hallucinations (61). Our findings also suggest that NE manipulation can potentially affect the positive symptoms associated with schizophrenia (43, 44), with the long-term study of adjunctive mirtazapine in patients with schizophrenia showing it improved PANSS positive scores (43), although, this may also be related to modulation of several 5-HT receptors (62). The effectiveness of mirtazapine in schizophrenia is supported by a preclinical study that showed that mirtazapine enhanced the AP activity of haloperidol in an apomorphine-induced climbing test in mice (63). Mirtazapine acts as an antagonist of α_2_-ARs, as well as serotonin 5-HT_2_ and 5-HT_3_ receptors (30), so that when used in combination with a first generation AP such as the D_2_ receptor antagonist haloperidol, the overall pharmacological profile resembles that of clozapine (43), which was reported to be superior to haloperidol for treating positive symptoms of schizophrenia (64). These findings are also supported by the pharmacological properties of brexpiprazole, a partial agonist at serotonin 5-HT_1A_ and DA D_2_ receptors, and a potent antagonist at serotonin 5-HT_2A_ and NE α_1B/2C_ receptors, all at similar potencies (36). Studies with brexpiprazole, recently approved for the treatment of schizophrenia, have demonstrated AP activity in rat models of the positive psychotic symptoms of schizophrenia (65), as well as significantly improved PANSS positive scores in patients with schizophrenia (37).

### NE Effects on Cognitive Systems

Compromised ability to think or concentrate is one of the diagnostic features of MDD. An imaging study showed that hyperactivity of the frontal and prefrontal cortices, areas of the brain innervated by noradrenergic neurons, is required for depressed patients to complete a working memory task at a level similar to healthy controls (66). Several cognitive processes, including attention and working memory, have been shown to be linked to NE activity (5). Indeed, cognitive defects in chromosome 22q11 deletion syndrome are associated with the abnormal function of several neurotransmitters, including NE (52). Furthermore, clonidine may exert dual activity by engaging inhibitory presynaptic α_2_-ARs, and therefore suppressing NE activity, while at the same time directly stimulating prefrontal cortex postsynaptic α_2_-ARs (39). Our review identified a study in which clonidine suppressed memory consolidation in patients with MDD and healthy controls (40). This role of α_2_-ARs in learning and memory consolidation is supported by preclinical studies in rats (67, 68), although Kuffel et al. (40) found no effect of noradrenergic blockade on working memory in patients with MDD or healthy participants. This is in contrast to an infusion study in which α_2_-AR agonists applied directly to the prefrontal cortex of monkeys and rats resulted in improved working memory performance (69). In addition, it is thought that the α_2A_-AR subtype may be the most important for cognitive improvement (5), as the α_2A_ selective agonist guanfacine is more effective at improving working memory without side effects than clonidine, which also acts at α_2B_- and α_2C_-ARs (70). High concentrations of NE have also been shown to reduce working memory function through actions at α_1_-ARs; indeed, stress-induced cognitive deficits have been shown to be blocked by infusion of an α_1_-AR antagonist into the prefrontal cortex of rats (71). Therefore, the balance between the activation of α_2A_-ARs and reducing the activity of α_1_-ARs may be a factor for consideration when designing pharmacological agents for the improvement of cognitive functions in MDD.

Recent studies have indicated a role for α_1_- and α_2_-ARs in regulation of cognitive processes, where α_2_-AR stimulation may ameliorate working memory deficits and α_1_-AR agonism may enhance sustained attention (72). Preclinical studies point to a differential role for subtypes of α_1_-ARs in cognition. While activation of α_1A_-ARs may have a positive impact on cognition and neurogenesis, α_1B_-mediated signaling may be detrimental to cognition and promote apoptosis (73, 74). Furthermore, while α_2A_-AR activation mediates improvement in working memory following guanfacine treatment (75), activity at α_2C_-ARs may also mediate stress-related psychiatric diseases such as MDD and anxiety disorders (76). Furthermore, α_2C_-AR antagonism may be associated with a pro-cognitive effect, in addition to ameliorating anxiety and depression (77). Thus, selective modulation of α-AR subtypes may be advantageous in therapy of MDD. This is indirectly supported by the pharmacological profile of brexpiprazole, which has antagonist activity at both α_1B_- and α_2C_-ARs (36), and has been shown to be an efficacious adjunctive treatment for patients with MDD (38).

In schizophrenia, defects in the early sensory filter mechanisms are thought to result in cognitive fragmentation, and ultimately hallucinations and delusions (78). Reduced filtering of sensory and sensorimotor information, demonstrated by both a reduced P50 suppression or reduced pre-pulse inhibition of the startle reflex, was corrected by activating α_2_-ARs with clonidine (39, 42). Other evidence from our search showed that mirtazapine increased mental speed and attention control in schizophrenia (44), which may be related to its activation of α_2_-ARs.

Glutamate has been shown to be crucial for cognitive processing, and pharmacological agents that modulate glutamate transmission, such as ketamine, have demonstrated ADT-like properties (14). Our search results suggested that glutamate transporter gene expression was reduced in LC astrocytes in postmortem brain tissue of patients with MDD (3). LC–NE activity is modulated by glutamatergic input, and so changes in glutamate levels could also indirectly impact the NE signaling. Elsewhere, increased glutamate levels have been reported in the occipital cortex, prefrontal cortex, and plasma of patients with MDD (79–81). Therefore, the benefit of glutamate modulators may in part be due to indirect actions on NE activity.

### Effects of NE on Other RDoC Systems

There was little direct evidence from our review to support a role of NE and α-ARs in the positive valence systems, systems for social processes, or arousal/regulation systems associated with MDD and schizophrenia. However, other studies outside the selection criteria for this review provided some data. Negative symptoms of schizophrenia, such as flat affect and poverty of speech, can be categorized into the social process RDoC system (13). Clinical studies have shown that mirtazapine enhanced the effects of haloperidol, risperidone, and clozapine on negative symptoms (82–84), which support the identified study that showed adjunctive mirtazapine improved PANSS negative scores (43).

Disruptions in sleep cycles and circadian rhythms are common in patients with MDD (85). Moreover, genetic studies have suggested that patients with MDD have both a phase shift of rhythms and a disrupted regulation of circadian genes (86). Since NE neurons project to the hypothalamus, which contains the suprachiasmatic nucleus—the master pacemaker, which coordinates rhythms throughout the brain and body (85), it is possible that NE has a role in modulating these effects. As reviewed elsewhere, imaging studies provide evidence of sleep disruption due to excessive arousal in MDD, while biochemical studies point to an association between elevated NE levels and decreased sleep efficiency (87). Thirty-hour sampling of CSF samples from depressed patients has found elevated NE relative to healthy controls around the clock (88).

### Predicting Response to Treatment

When considering individual patient needs, biomarkers which aid in the diagnosis of patients or predict responses to treatment are valuable for successful therapeutic intervention. MDD and schizophrenia were originally thought to be diseases located entirely in the CNS, although recent hypotheses and findings suggest that the pathophysiology of these diseases can be shaped by peripheral endocrine, autonomic, immune, and gut-brain events (24, 89, 90). Several metabolic studies have attempted to identify potential biomarkers for MDD and schizophrenia by assessing changes in enzymatic activity in plasma and saliva. Baseline levels of MHPG in saliva were shown to positively correlate with a high Beck Depression Inventory score in men (19), which provides support to the findings that MHPG concentrations in CSF were correlated with a lifetime burden of mood swings (18). In addition, plasma MHPG has been shown to correlate with negative symptoms and cognitive impairment during early-stage schizophrenia (91).

A patient’s NE metabolites or genetic profile may also help to predict how well they will respond to different treatments, not only with regards to improving symptoms, but also the emergence of adverse events. A study found that patients with MDD that responded to mirtazapine or SSRI treatment had higher baseline saliva MHPG concentrations, although this did not reach statistical significance in patients receiving mirtazapine alone, or when it was used as adjunctive therapy with SSRIs (47). Similarly, risperidone, which acts as an antagonist of α_1_- and α_2_-ARs (as well as other receptors), was shown not to affect NE, vanillylmandelic acid, or MHPG in the plasma or urine of patients with schizophrenia (35).

Genetic studies identified here suggested that differences in the gene which codes for α_1A_-AR are associated with weight gain (50) and metabolic abnormalities (49) during AP treatment of schizophrenia. However, there are many factors that affect weight gain in patients with schizophrenia, and attribution of this adverse reaction to a single receptor type is inappropriate. For example, it has been shown that antagonism of the DA D_2_ receptor has both an effect on eating, mediated by 5-HT_2C_ receptors, and disinhibition of prolactin secretion, which has an impact on lipid and glucose metabolism (92).

The atypical AP olanzapine and the SSRI fluoxetine are often used in combination in patients with treatment-resistant MDD. Previously, olanzapine and fluoxetine have been shown to have a synergistic effect on elevation of NE and DA levels in the rat prefrontal cortex (93), which may be responsible for some of the efficacy of this combination therapy. Therefore, genetic variants in the NET identified in patients with MDD who were not initially responsive to fluoxetine alone may be a marker to predict improvements in these patients after switching to olanzapine–fluoxetine combination (45). Quetiapine was also shown to occupy NET in healthy patients at doses that produce antidepressive effects. This may explain the unique broad spectrum efficacy of quetiapine therapy in psychiatric disorders (55).

The higher density of α_2_-ARs in both ADT-free and antidepressant-treated patients with depression compared with matched controls suggests that these receptors are resistant to the observed downregulation of β_1_-ARs by ADTs (48). In a preclinical study in rats, treatment with the SSRI citalopram did not result in any adaptive changes in LC neurons, but did indirectly enhance the inhibitory effect of NE on neurons in the LC (94). The activation of inhibitory α_2_-ARs resulting from elevation of NE concentrations in the synaptic cleft may underlie this effect (94). Although, desensitization of these receptors may also have occurred, as previously observed in animal studies (95, 96). Furthermore, preclinical evidence also suggests that intact α_1_-AR-mediated signaling may be necessary for SSRI induced 5-HT elevation (97).

Both MDD and schizophrenia have been associated with immune abnormalities and dysfunctional inflammatory signaling (98, 99). Elevated peripheral inflammation may precipitate aberrant CNS inflammatory signaling with subsequent disruption of monoamine and glutamate transmission, in part by inappropriately activating brain microglia (100). Both α- and β-ARs have a key role in regulating macrophage and microglia activity (101, 102). Thus, compromised NE transmission may promote a pro-inflammatory phenotype consistent with depressive psychopathology. Intriguingly, a recent study reported a preferential response to an SSRI (escitalopram) in patients with lower levels of an inflammatory marker [C-reactive protein (CRP) <1 mg/mL], compared with depressed patients with higher inflammation (CRP > 3 mg/mL) who had a robust response to a predominantly noradrenergic ADT (nortriptyline) (103). Therefore, one may speculate that noradrenergic ADTs exert some of their therapeutic effect by attenuating excessive inflammatory signaling.

### Limitations

The objective of our systematic review was to determine the role of NE and α-ARs in the pathophysiology of MDD and schizophrenia using evidence from clinical reports. In order to achieve this, a number of specific search terms were implemented, including the use of “alpha adrenergic receptor(s)” as a limiter in the MeSH search-term string. Therefore, any reports that did not contain this term were excluded, even though they may indirectly support the role of NE activity at α-ARs in MDD or schizophrenia. It should also be noted that in order to focus this literature review on recent data, the scope was restricted to publications between 2010 and 2015, which will limit our findings. Finally, it is a daunting challenge to attempt to link receptor activity to specific therapeutic effect, especially as many of the drugs discussed in this review have clinically relevant binding affinities for a number of different receptors, and additionally, these diseases are phenotypically, genetically, and biologically heterogeneous. The lack of a common pathophysiology or homogenous symptom presentation between all patients diagnosed with MDD or schizophrenia will impact the summary of effects linked to α-AR modulation. Moreover, it has been reported that females have a larger LC than males, comprising nearly 3,000 more neurons, which could impact their capacity for NE production and release (104). Given these neurobiological differences, we suggest that gender should be considered as a factor in future clinical studies.

## Conclusion

Preclinical evidence suggests that targeting NE α-ARs is beneficial for the treatment of both MDD and schizophrenia. However, the complex and heterogeneous nature of both of these diseases means that interpretation of clinical findings is not always straightforward. Using a systematic approach, we have provided indirect evidence from recent clinical studies and other reports in humans that support the concept that, in general, NE and α-ARs, and drugs that act through these receptors, have an important role in the negative valence systems and cognitive systems related to MDD and schizophrenia. However, there is no direct clinical evidence for the specific effects of α-ARs, likely due to a lack of agents that have proven differential subtype affinity. Moreover, no direct clinical comparisons have been made between therapies that have α-AR activity. Therefore, advantages of the interactions at these receptors are theoretical in the clinical situation, supported only by evidence from preclinical experiments. In addition, due to both the fact that clinical trials have not been designed to determine if there are differences in efficacy between different symptom domains, and the added complexity of the heterogeneity of these diseases across populations of patients, the effects of targeting distinct α-ARs on specific symptom domains are yet to be determined. These points should be considered when studying future medications.

It is worth noting that genetic studies may suggest that candidate genes could have predictive properties enabling their use as putative biomarkers for disease in target populations. However, there are multiple influences on MDD and schizophrenia, and blanket SNP studies conducted to date have not revealed any specific relationships with NE. The limited number of studies identified here suggests that more up-to-date clinical investigations, designed to tease apart the contribution of α-ARs and their subtypes to the pathophysiology of MDD and schizophrenia, are required.

## Author Contributions

VM provided substantial contributions to the manuscript in leading the design of the literature search, defining the inclusion and exclusion criteria, screening of the articles, providing critical reviews and revisions for each of the drafts, and approving the final version for submission. AE, KG, SO, and RD provided substantial contributions to the manuscript in closely participating in designing the literature search, defining the inclusion and exclusion criteria, screening of the articles, providing critical reviews and revisions for each of the drafts, and approving the final version for submission. VM, AE, KG, SO, and RD agreed to be accountable for all aspects of the work in ensuring that questions related to the accuracy and integrity of the work are appropriately investigated and resolved.

## Conflict of Interest Statement

AE and KG are employees of Lundbeck LLC. SO and RD are employees of Otsuka Pharmaceutical Development and Commercialization, Inc.
